# Primary Care Physician Recognition and Documentation of Depressive Symptoms Among Chinese and Latinx Patients During Routine Visits: A Cross-Sectional Study

**DOI:** 10.1089/heq.2020.0104

**Published:** 2021-04-26

**Authors:** Maria E. Garcia, Ladson Hinton, Steven E. Gregorich, Jennifer Livaudais-Toman, Celia P. Kaplan, Mitchell Feldman, Leah Karliner

**Affiliations:** ^1^ Center for Aging in Diverse Communities, University of California, San Francisco, California, USA.; ^2^ Multiethnic Health Equity Research Center, Division of General Internal Medicine, Department of Medicine, University of California, San Francisco, California, USA.; ^3^ Division of General Internal Medicine, Department of Medicine, University of California, San Francisco, California, USA.; ^4^ Department of Psychiatry and Behavioral Sciences, University of California, Davis, California, USA.

**Keywords:** depressive symptoms, depression recognition, depression treatment, language barriers, limited English proficiency

## Abstract

**Purpose:** Asian and Latinx individuals have a high burden of untreated depression. Under-recognition of depressive symptoms may contribute to existing disparities in depression treatment. The objective of this cross-sectional study was to determine whether physicians recognize and treat depressive symptoms for Chinese and Latinx patients during routine primary care visits.

**Methods:** We analyzed data from 1171 Chinese and Latinx patients who were interviewed within 1 week after a primary care visit in a large academic practice, which had not yet implemented universal depression screening. We included participants with depressive symptoms (defined as a Patient Health Questionaire-2 score ≥3) and no prior history of depression (*N*=118). We investigated whether patients perceived having a mental health need in the prior year and conducted chart reviews to assess provider recognition of depressive symptoms, defined as documentation of symptoms, antidepressant initiation, or mental health referral within 30 days of the visit. We further examined differences by race/ethnicity and language preference.

**Results:** Among the 118 patients with depressive symptoms and no prior depression diagnosis (mean age 68), 71 (61%) reported a mental health need in the prior 12 months; however, providers recognized depressive symptoms in only 8/118 patients (7%). The number of patients with recognized symptoms was small across race/ethnicity and language preference groups and we found no significant differences.

**Conclusion:** Physicians recognized and documented depressive symptoms for 1 in 10 Chinese and Latinx patients during routine primary care visits. Targeted efforts are needed to address under-recognition of symptoms and improve depression care for these populations.

## Introduction

Depression is associated with poor mental and physical health outcomes and earlier mortality.^1–7^ While depression treatment can improve depressive symptoms, as well as management of chronic health conditions, many patients continue to have undiagnosed and untreated depression.^8–11^ Experts have called for the aggressive detection and treatment of mental health symptoms, particularly during times of increased stressors, such as the COVID-19 pandemic.^12–14^ Yet prior studies suggest that physicians fail to recognize depressive symptoms in a large number of their patients.^11,15–17^

Prior studies have found that racial/ethnic minority patients who are clinically depressed are less likely to be diagnosed and treated for their depression compared to non-Hispanic whites.^15,18–28^ Asian and Latinx individuals are more likely to have untreated depression.^18,19,29,30^ Yet it is unknown to what extent physicians recognize depressive symptoms among Chinese and Latinx patients without a prior diagnosis of depression and how this may contribute to mental health treatment disparities. Without recognition, physicians cannot begin to address depressive symptoms, representing a missed opportunity for diagnosis and leading to a potential gap in treatment for patients with depressive symptoms. Primary care physicians are uniquely poised to detect, refer, and/or treat patients with depressive symptoms.

A better understanding of gaps in symptom recognition among Chinese and Latinx patients will help to improve depression screening and management in primary care. The primary aim of this study was to investigate the proportion of Chinese and Latinx patients with current depressive symptoms, whose symptoms were recognized during a routine primary care visit in the absence of universal depression screening. A secondary goal was to examine differences in depression symptom recognition by race/ethnicity and preferred language.

## Methods

### Sample

In this secondary data analysis, we analyzed data from the Language Access System Improvement (LASI) study, which has been described previously.^31–34^ LASI was designed to evaluate the effects of simultaneously increasing access to professional interpreters and certifying bilingual physicians' language skills on communication and clinical outcomes in primary care. LASI recruited patients from an academic primary care practice, which serves an ethnically, linguistically, and socioeconomically diverse population of over 25,000 adult patients in San Francisco.

Patients were eligible for LASI if they were ≥40 years; self-identified as Latinx or Chinese (the largest populations with limited English proficiency [LEP] in our practice); preferred English, Spanish, Cantonese, or Mandarin; received primary care in the practice; and were able to participate in a telephone interview. In this practice, patients with LEP tend to be older than the general English-speaking population. To avoid confounding by age, LASI purposively sampled older English speakers for a comparison group, resulting in an older mean age of study participants. Bilingual/bicultural research assistants conducted telephone interviews in the patient's preferred language within 1 week of a primary care visit (the “index visit”). The majority of LASI participants were seen by primary care physicians, with a smaller number seen by primary care nurse practitioners or medicine residents between January 2016 and July 2017. This study was conducted before the implementation of universal depression screening in the primary care practice.

### Current depressive symptoms

We defined current depressive symptoms using the participant's response on the post-visit telephone survey to the Patient Health Questionnaire-2 (PHQ-2). The PHQ-2 is a well-validated and widely used clinical measure to screen for current depressive symptoms, which includes two questions, “In the two weeks before coming to the clinic, how often were you bothered by any of the following problems? (1) Little interest or pleasure in doing things? (2) Feeling down, depressed, or hopeless?” Possible answers to each question include “not at all,” “several days,” “more than half the days,” and “nearly every day,” or decline to answer, with total possible scores from 0 to 6. Participants were categorized as having current depressive symptoms if they had a PHQ-2 score ≥3 (sensitivity of 61–87% and specificity of 78–92% for major depression^35–38^). The PHQ-2 has been validated in both Spanish and Chinese with similar sensitivity and specificity among ethnically Latinx and Chinese populations as among non-Hispanic whites.^39–43^ In our study, physicians were unaware of the PHQ-2 results at the time of visit as the measure was collected by research assistants during the telephone survey within 1 week after the index clinical visit.

Given that those with pre-existing depression could have already discussed their symptoms with their physician, or that the current elevated PHQ-2 score represented an improvement in symptom score, we limited our sample to those participants who were likely experiencing new symptoms. We excluded those with a prior history of depression listed on the electronic medical record (EMR) problem list before the index visit or who had a diagnosis of depression entered at a previous outpatient encounter (ICD-10 codes: F20.4, F31.3–F31.5, F32.x, F33.x, F34.1, F41.2, and F43.2).

### Study sample

The LASI study identified 2359 potentially eligible participants; 662 (28%) were unreachable for the telephone interview. Among 1697 reached, 1181 agreed to participate (69.6% participation rate). Of these, 1171 participants (435 EP and 736 LEP) completed a PHQ-2 (99%). Two hundred twenty-one participants (19%) had current depressive symptoms. Of those, 118 participants (53%) had no history of depression and were included in this analysis.

### Physician recognition of depressive symptoms

We defined physician recognition of depressive symptoms as a composite outcome, including any of the following: the physician documented a discussion of depressive symptoms in the clinical visit note, coded for depression (ICD-10 codes as above), initiated an antidepressant, or referred the patient to mental health services (including referral to social work for depression, psychiatry, or behavioral health services) at the index visit or at a follow-up visit within 30 days (a reasonable time frame to reassess for depressive symptoms^44^). Data were obtained from the EMR and through chart reviews. We determined suicide risk assessment through chart review.

### Race/ethnicity and English language proficiency

Latinx and Chinese ethnicity as well as gender were defined by self-report in the post-visit telephone survey. Participants were classified as having English proficiency or LEP based on a validated measure^45^ of English language proficiency that includes patient-reported preferred language for discussing health care and the patient's response to the U.S. Census question, “How well do you speak English?”^46^ Response options include “very well,” “well,” “not well, and “not at all.” Participants who spoke English “very well” or who spoke English “well” and preferred to discuss their health care in English were classified as having English Proficiency.^31^ All others were classified as having LEP.

### Prior year perceived mental health need

Patient-reported perceived mental health need in the prior year was measured using a question adapted from the 2013 to 2014 California Health Interview Survey^47^ and the 2002–2003 National Latino and Asian American Study^48^ (CHIS and NLAAS; population-based surveys): “During the past 12 months, did you think you needed help for emotional or mental health problems, such as feeling sad, blue, anxious, or nervous?” As has been defined in prior studies, an affirmative response indicated not only that participants had experienced mental health problems but also that they felt they needed help in addressing these problems.^21,49^

### Other covariates

Additional demographics and participant characteristics included patient-reported age, education, health literacy,^50,51^ and insurance type (private, Medicare, or MediCal). Participant clinical characteristics and health service utilization (from chart review and EMR queries) included Elixhauser comorbidities,^52,53^ whether the participant had been in the practice for at least 1 year, the number of primary care practice visits in the prior year, the number of problems listed in the assessment and plan of the index visit, and whether the patient was seen by their own Primary Care Physician (PCP) or another physician at the index visit. We additionally categorized the index visits for patients with LEP as language concordant, discordant (either professionally interpreted or nonprofessionally interpreted), and partially concordant (see Table 1 footnote).

**Table 1.** Demographics for Chinese and Latinx patients with current depressive symptoms (Patient Health Questionnaire-2 score ≥3) and no history of depression (*N*=118)

	Chinese (N=78; 66%), N (%)	Latinx (N=40; 34%), N (%)	p
Age, years, mean±SE	70±2.0	65±2.1	0.11
LEP	53 (68)	21 (53)	0.10
Women	55 (71)	28 (70)	0.95
Education (*N*=117^a^)			0.36
Less than high school	35 (45)	18 (46)	
High school diploma	16 (21)	12 (31)	
AA or some college	7 (9)	4 (10)	
College degree or higher	20 (26)	5 (13)	
Adequate health literacy^b^	47 (60)	22 (55)	0.56
Patient in clinic ≥1 year	72 (92)	39 (98)	0.25
Number of clinic visits in past year, mean±SE	3.3±0.28	3.6±0.44	0.57
Comorbidities (count),^c^ mean±SE	2.6±0.26	2.6±0.32	0.88
Insurance status			0.37
Private	12 (15)	10 (25)	
Medicare	55 (71)	23 (58)	
MediCal	11 (14)	7 (18)	
Patient seen by PCP at index visit	54 (69)	31 (78)	0.43
Visit language concordance^d^			0.35
English concordant	25 (32)	19 (48)	
Non-English language concordant	16 (21)	7 (18)	
Discordant, professional interpreter	26 (33)	7 (18)	
Discordant, *ad hoc* interpreter	6 (8)	6 (15)	
Partially concordant^d^	5 (6)	1 (3)	
Number of problems addressed at the index visit, mean±SE	4.8±0.56	4.3±0.35	0.37

Percents may not equal 100% due to rounding.

^a^ One Latinx participant did not provide educational attainment.

^b^ Health literacy was determined using a single, validated question, “How confident are you filling out medical forms by yourself?”

^c^ Diagnoses were included from the Elixhauser comorbidities count (in alphabetical order): AIDS, Alcohol abuse, Anemia, Cardiac arrhythmias, Chronic kidney disease, Chronic pulmonary disease, Coagulopathy, Congestive heart failure, Coronary artery disease, Diabetes, Drug abuse, Hypertension, Hypothyroidism, Liver disease diagnosis, Lymphoma, Metastatic cancer, Fluid or electrolyte disorder, Neurological disorder, Non-metastatic cancer, Obesity, Paralysis, Peptic ulcer disease, Peripheral vascular disorder, Pulmonary circulation disorder, Renal failure diagnosis, Rheumatoid arthritis, Valvular disease, and Weight loss.

^d^ We categorized the index visits for patients with LEP as language concordant (in English or non-English language), discordant-professionally interpreted, and discordant-not-professionally interpreted, and partially concordant. Visits were considered partially concordant if (1) physician reported “none,” “poor,” or “fair” skills in patient's preferred non-English language and patient reported speaking English “Well,” (2) physician reported “good,” “very good,” or “excellent” skills in patient's preferred non-English language, but failed language test in that language, or (3) physician reported “good,” “very good,” or “excellent” skills in patient's preferred non-English language, but they did not take the language test and had 3 or more patients give them an average rating of their skills as less than “very good” or “excellent.”

AA, Associate of Arts Degree; LEP, limited English proficiency; PCP, Primary Care Physician; SE, standard error.

### Statistical analysis

We computed the proportion of participants with current depressive symptoms with no prior history of depression, who had physician-recognized symptoms. We further examined each component of the composite outcome: documented discussion of depressive symptoms, initiation of an antidepressant, and referral for mental health treatment at the initial visit at the index visit or any visit in the clinical practice within 30 days. We examined bivariate relationships of covariates with physician recognition of depression symptoms, with a plan to conduct a multivariate logistic regression examining the association of race/ethnicity and language preference with our outcome.

Given that initial presentation of depression may include nonspecific mood symptoms, we then conducted a sensitivity analysis in which we included physician recognition of other mental health symptoms in our composite outcome. This included documentation of anxiety, bereavement, or trauma discussion or a mental health referral at the initial visit or a subsequent visit within 30 days of the index visit.

All analyses accounted for clustering of patients in the care of a physician. Stata version 14.2 was used for all calculations (StataCorp LP, College Station, TX). This study was approved by the University of California, San Francisco (UCSF) Institutional Review Board (IRB).

## Results

### Sample characteristics and prevalence of current depressive symptoms

The 118 participants with current depressive symptoms and no history of depression tended to be older than 65 years (mean age 68), included majority women (70%), most had LEP (63%), had adequate health literacy (58%), were seen by their PCP during the index visit (72%), and were seen in language-concordant visits in English or a non-English language (57%; Table 1).

### Prior year perceived mental health need

Seventy-one participants (61%) perceived a mental health need in the prior year. Chinese participants were more likely to perceive a mental health need than Latinx participants (71% vs. 40%; *p*<0.001).

### Physician recognition of depressive symptoms

**FIG. 1.**
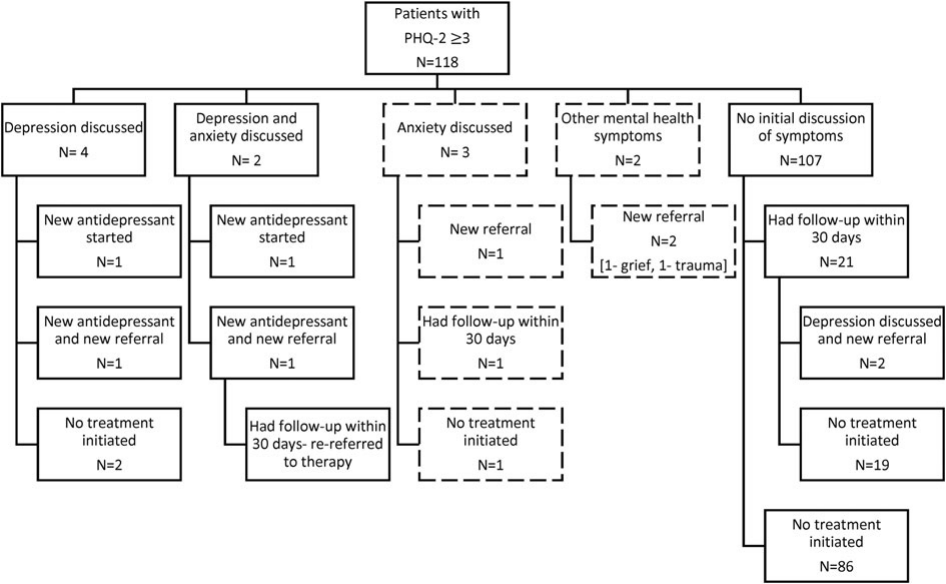
Discussion and treatment of current depressive symptoms (PHQ-2 score ≥3) and other mental health symptoms for Chinese and Latinx patients with no prior history of depression (*N*=118). PHQ-2, Patient Health Questionnaire-2.

In total, only eight unique participants (7% of those with PHQ-2 score ≥3) had a documented discussion of depression (Fig. 1). For these participants, physicians initiated an antidepressant for two participants (25%), both antidepressant and referral for two participants (25%) at the index visit, and placed a referral for two participants at a subsequent visit (25%). Two participants (25%) had a discussion about depressive symptoms, but no treatment was initiated. Out of the eight participants, four had negative suicide risk assessment (50%). Twenty-three participants (19%) with depressive symptoms had a follow-up visit with their primary care physician within 30 days of the index visit.

In the sensitivity analysis, including physician recognition of other mental health symptoms, a total of 13 (11%) unique participants discussed any mental health symptoms (8 depression, 3 anxiety, 1 grief, and 1 physical and emotional trauma). We found no significant difference in recognition and treatment initiation by race/ethnicity (Chinese vs. Latinx) or English proficiency (LEP vs. EP), although the number of participants with recognized symptoms was small. The number of patients with LEP with recognized symptoms was too small to evaluate for differences by language concordance or professional interpretation.

## Discussion

Only a small number of patients with no history of depression had their depressive symptoms recognized and documented during a routine primary care visit. Even when we included physician recognition of other mental health symptoms (anxiety, grief, and trauma), in our definition of physician-recognized depressive symptoms, only 1 in 10 participants with depressive symptoms was recognized.

These findings are consistent with prior studies of poor provider recognition of current depressive symptoms in other primary care samples.^15–17^ In another study using EMRs, only 12% of patients with moderate to severe depressive symptoms (based on a PHQ-8 score ≥10) were recognized over the course of a year.^15^ In another prospective cohort study of primary care patients followed over 5 years, only 56% had their symptoms of major depression at baseline (diagnosed by psychiatric interview) recognized and treated over the study period.^17^ However, many of the prior studies included few, if any, Asian and Latinx^21,54,55^ patients. Poor recognition of depressive symptoms may contribute to the mental health disparities observed for ethnically Chinese and Latinx patients.^18,21,26–30,56^ In addition, older racial and ethnic minority patients, such as those included in our study, are at higher risk of poor recognition of depressive symptoms. Older adults may be more likely to have comorbidities causing competing demands in a primary care visit, be less likely to perceive a need for care, have limited knowledge and availability of affordable mental health services, and have difficulty arranging transportation.^21,54,55^

The COVID-19 pandemic has made recognition and treatment of depressive symptoms increasingly critical. Due to the COVID-19 pandemic and subsequent shelter in place orders, there has been self-imposed isolation and loneliness, increased financial strain and unemployment rates, and a reduction in services, as well as the fear of becoming infected. This has placed individuals at increased risk for mental health symptoms.^12,14^ Moreover, the pandemic has disproportionately affected racial and ethnic minorities in the United States.^57,58^ To address the likely growth in service need for depression and anxiety, targeted efforts to recognize symptoms and engage racial and ethnic minorities in mental health treatment will be needed.

Barriers to recognition of depressive symptoms in primary care are multifactorial and include patient, physician, and system factors. Patients may present or focus on somatic complaints rather than classic mood symptoms, under-report or minimize depressive symptoms, or reject the diagnosis of depression due to stigma or cultural norms.^59–62^ Physicians may be equally concerned about patient stigma regarding a mental health diagnosis and may avoid discussions about mental health symptoms or treatment.^63,64^ Physician characteristics may influence comfort with discussing and addressing depressive symptoms, such as gender, racial, and ethnic concordance, years of practice, training background, implicit bias, setting, and familiarity with patients.^63–66^ Patient-physician communication factors, such as language barriers, may further contribute to missing depression diagnoses.^30,49,63,67^ In addition, physicians often have competing demands and time pressures during routine primary care visits, which may be further compounded for older adults with multiple comorbidities.^21,54^ Systems factors include pressures on physicians to maintain productivity, limitations in mental health coverage for patients, limited access to specialty care, inadequate availability, and limited continuity of care, as well as few protocols and resources in place to systematically detect and manage patients with depression in many primary care settings.^68,69^ The extent to which these factors played a role in the poor recognition of depressive symptoms in this sample of Chinese and Latinx primary care patients is unknown and should be further examined in future studies.

It is possible that physician “recognition” of depressive or other mental health symptoms may be more closely correlated with clinical action. In other words, physicians may recognize mental health symptoms, but not document these if they do not meet their threshold for clinical action. It is possible that some of the patients with elevated depressive symptoms on the PHQ-2, who did not receive a depression diagnosis or treatment/referral, were “false positives” for depression that does not meet a clinical threshold, or that due to the timing of our research study (asking patients about depressive symptoms within a week of the index visit), we may be misclassifying participants with less than 2 weeks of symptoms when seen by physicians. However, the high participant-perceived mental health need in this group suggests that many did have depression or another mental health condition at some point in the prior 12 months. Also, a positive response to the mental health need measure used in this study suggests that these patients wanted their mental health symptoms recognized and acknowledged, although it is possible that this mental health need had resolved before the index visit.

The small number of patients with physician-recognized depressive symptoms limited our ability to investigate the individual contribution of factors that may affect physician recognition of depressive symptoms, such as language concordance, gender concordance, or continuity of care with physician. For participants with LEP, it remains unclear whether professional interpretation impacts recognition of depressive symptoms or if language concordance improves recognition.

Our study has limitations. The primary limitation is that we used a depression screening instrument outside of a clinical visit; patients may be more likely to disclose depressive symptoms in a research setting than a clinical setting, especially to bilingual, bicultural research assistants. In addition, we do not have a clinical assessment of depression as a gold standard or information about the severity of participants' depressive symptoms. It may be that those participants whose physicians did not recognize their symptoms by our definition only had minor depressive symptoms that may resolve over time without treatment; however, our inclusion of documentation of symptoms in any part of the clinical note rather than only a diagnosis casts a broad net to capture these patients as well. It is also possible that some of those who screened positive for depression did so due to other physical health conditions rather than depression, although the PHQ-2 is specific to mood and anhedonia rather than physical symptoms.^11^ Physicians may also be accurately assessing the presence of another mental health condition or its co-existence with depressive symptoms, although including recognition of these symptoms in our definition of recognition of depressive symptoms only increased the percent of participants with recognized symptoms from 7% to 11%. We may also be including some individuals with a history of depression not documented in the EMR, and thus, despite our best efforts, the depressive symptoms may not be a new finding for physicians.

## Conclusion

Primary care physicians may be failing to recognize and address current depressive symptoms in the large majority of their Chinese and Latinx patients (with and without language barriers) with no prior history of depression. As the COVID-19 pandemic has disproportionately impacted racial and ethnic minorities, and the number of individuals with mental health symptoms grows, this represents a potential missed opportunity and could lead to a further gap in treatment for Chinese and Latinx patients with depressive symptoms. While systematic depression screening may identify patients whose symptoms would otherwise go unrecognized, further training is also needed to increase provider detection and management of depression, particularly for racial and ethnic minority groups. It will be important to study barriers in the context of universal screening and identify additional supports needed to address depression treatment barriers for these vulnerable populations.

## Abbreviations Used

AA: Associate of Arts Degree
EMR: electronic medical record
LASI: Language Access System Improvement
LEP: limited English proficiency
PCP: Primary Care Physician
PHQ-2: Patient Health Questionnaire-2
SE: standard error

## References

[1] Carney RM, Freedland KE, Steinmeyer B, et al. Depression and five year survival following acute myocardial infarction: a prospective study. J Affect Disord. 2008;109:133–138 18191208 10.1016/j.jad.2007.12.005 PMC2491401

[2] Hofmann M, Köhler B, Leichsenring F, et al. Depression as a risk factor for mortality in individuals with diabetes: a meta-analysis of prospective studies. PLoS One. 2013;8:e79809 24278183 10.1371/journal.pone.0079809 PMC3836777

[3] Egede LE. Major depression in individuals with chronic medical disorders: prevalence, correlates and association with health resource utilization, lost productivity and functional disability. Gen Hosp Psychiatry. 2007;29:409–416 17888807 10.1016/j.genhosppsych.2007.06.002

[4] Egede LE. Diabetes, major depression, and functional disability among U.S. adults. Diabetes Care. 2004;27:421–428 14747223 10.2337/diacare.27.2.421

[5] Egede LE, Zheng D, Simpson K. Comorbid depression is associated with increased health care use and expenditures in individuals with diabetes. Diabetes Care. 2002;25:464–470 11874931 10.2337/diacare.25.3.464

[6] de Groot M, Anderson R, Freedland KE, et al. Association of depression and diabetes complications: a meta-analysis. Psychosom Med. 2001;63:619–630 11485116 10.1097/00006842-200107000-00015

[7] Black SA, Markides KS, Ray LA. Depression predicts increased incidence of adverse health outcomes in older Mexican Americans with type 2 diabetes. Diabetes Care. 2003;26:2822–2828 14514586 10.2337/diacare.26.10.2822

[8] Katon WJ, Lin EHB, Von Korff M, et al. Collaborative care for patients with depression and chronic illnesses. N Engl J Med. 2010;363:2611–2620 21190455 10.1056/NEJMoa1003955 PMC3312811

[9] Ramanuj P, Ferenchick EK, Pincus HA. Depression in primary care: part 2—management. BMJ. 2019;365:1835 10.1136/bmj.l835 30962249

[10] Fuentes D, Aranda MP. Depression interventions among racial and ethnic minority older adults: a systematic review across 20 years. Am J Geriatr Psychiatry. 2012;20:915–931 22828202 10.1097/JGP.0b013e31825d091a PMC3479358

[11] Egede LE. Failure to recognize depression in primary care: issues and challenges. J Gen Intern Med. 2007;22:701–703 17370030 10.1007/s11606-007-0170-z PMC1852925

[12] Galea S, Merchant RM, Lurie N. The mental health consequences of COVID-19 and physical distancing: the need for prevention and early intervention. JAMA Intern Med. 2020;180:817–818 32275292 10.1001/jamainternmed.2020.1562

[13] Force (USPSTF) UPST. Screening for depression in adults: US preventive services task force recommendation statement. JAMA. 2016;315:380–387 26813211 10.1001/jama.2015.18392

[14] Pfefferbaum B, North CS. Mental health and the Covid-19 pandemic. N Engl J Med. 2020;383:510–512 32283003 10.1056/NEJMp2008017

[15] Hudson DL, Karter AJ, Fernandez A, et al. Differences in the clinical recognition of depression in diabetes patients: the Diabetes Study of Northern California (DISTANCE). Am J Manag Care. 2013;19:344–352 23781889 PMC3703822

[16] Cepoiu M, McCusker J, Cole MG, et al. Recognition of depression by non-psychiatric physicians—a systematic literature review and meta-analysis. J Gen Intern Med. 2008;23:25–36 17968628 10.1007/s11606-007-0428-5 PMC2173927

[17] Jackson JL, Passamonti M, Kroenke K. Outcome and impact of mental disorders in primary care at 5 years. Psychosom Med. 2007;69:270–276 17401055 10.1097/PSY.0b013e3180314b59

[18] Alegría M, Chatterji P, Wells K, et al. Disparity in depression treatment among racial and ethnic minority populations in the United States. Psychiatr Serv. 2008;59:1264–1272 18971402 10.1176/appi.ps.59.11.1264 PMC2668139

[19] Alegría M, Mulvaney-Day N, Woo M, et al. Correlates of past-year mental health service use among Latinos: results from the National Latino and Asian American Study. Am J Public Health. 2007;97:76–83 17138911 10.2105/AJPH.2006.087197 PMC1716237

[20] Alegría M, Mulvaney-Day N, Torres M, et al. Prevalence of psychiatric disorders across Latino subgroups in the United States. Am J Public Health. 2007;97:68–75 17138910 10.2105/AJPH.2006.087205 PMC1716243

[21] Sorkin DH, Pham E, Ngo-Metzger Q. Racial and ethnic differences in the mental health needs and access to care of older adults in california. J Am Geriatr Soc. 2009;57:2311–2317 19943830 10.1111/j.1532-5415.2009.02573.x

[22] Hinton L, Apesoa-Varano EC, González HM, et al. Falling through the cracks: gaps in depression treatment among older Mexican-origin and white men. Int J Geriatr Psychiatry. 2012;27:1283–1290 22383214 10.1002/gps.3779 PMC3560929

[23] Chung H, Teresi J, Guarnaccia P, et al. Depressive symptoms and psychiatric distress in low income Asian and Latino primary care patients: prevalence and recognition. Community Ment Health J. 2003;39:33–46 12650554 10.1023/a:1021221806912

[24] Tran LD, Ponce NA. Who gets needed mental health care? Use of mental health services among adults with mental health need in California. Californian J Health Promot. 2017;15:36–45 PMC5515380 28729814

[25] Sue S, Yan Cheng JK, Saad CS, et al. Asian American mental health: a call to action. Am Psychol. 2012;67:532–544 23046304 10.1037/a0028900

[26] Kim G, Kim M, Park S, et al. Limited English proficiency and trajectories of depressive symptoms among Mexican American older adults. Gerontologist. 2019;59:856–864 29688320 10.1093/geront/gny032 PMC6857694

[27] Sentell T, Shumway M, Snowden L. Access to mental health treatment by English language proficiency and race/ethnicity. J Gen Intern Med. 2007;22(Suppl 2):289–293 10.1007/s11606-007-0345-7 PMC2150610 17957413

[28] Cabassa LJ, Zayas LH, Hansen MC. Latino adults' access to mental health care: a review of epidemiological studies. Adm Policy Ment Health. 2006;33:316–330 16598658 10.1007/s10488-006-0040-8 PMC2551758

[29] Abe-Kim J, Takeuchi DT, Hong S, et al. Use of mental health-related services among immigrant and US-born Asian Americans: results from the National Latino and Asian American Study. Am J Public Health. 2007;97:91–98 17138905 10.2105/AJPH.2006.098541 PMC1716256

[30] Bauer AM, Chen C-N, Alegría M. English language proficiency and mental health service use among Latino and Asian Americans with mental disorders. Med Care. 2010;48:1097–1104 21063226 10.1097/MLR.0b013e3181f80749 PMC3135417

[31] Garcia ME, Hinton L, Gregorich SE, et al. Unmet mental health need among Chinese and Latino primary care patients: intersection of ethnicity, gender, and English proficiency. J Gen Intern Med. 2020;35:1245–1251 31667737 10.1007/s11606-019-05483-9 PMC7174511

[32] Roter DL, Gregorich SE, Diamond L, et al. Loss of patient centeredness in interpreter-mediated primary care visits. Patient Educ Couns. 2020;103:2244–2251 32819755 10.1016/j.pec.2020.07.028 PMC8454264

[33] Nouri S, Khoong EC, Lyles CR, et al. Addressing equity in telemedicine for chronic disease management during the Covid-19 pandemic. NEJM Catal Innov Care Deliv. 2020. [Epub ahead of print]; DOI: 10.1056/CAT.20.0123

[34] Pathak S, Gregorich SE, Diamond LC, et al. Patient perspectives on the quality of professional interpretation: results from LASI study. J Gen Intern Med. 2021. [Epub ahead of print]; DOI: 10.1007/s11606-020-06491-w PMC7845580 33515189

[35] Maurer DM. Screening for depression. Am Fam Physician. 2012;85:139–144 22335214

[36] Löwe B, Kroenke K, Gräfe K. Detecting and monitoring depression with a two-item questionnaire (PHQ-2). J Psychosom Res. 2005;58:163–171 15820844 10.1016/j.jpsychores.2004.09.006

[37] Arroll B, Goodyear-Smith F, Crengle S, et al. Validation of PHQ-2 and PHQ-9 to screen for major depression in the primary care population. Ann Fam Med. 2010;8:348–353 20644190 10.1370/afm.1139 PMC2906530

[38] Mitchell AJ, Yadegarfar M, Gill J, et al. Case finding and screening clinical utility of the Patient Health Questionnaire (PHQ-9 and PHQ-2) for depression in primary care: a diagnostic meta-analysis of 40 studies. BJPsych Open. 2016;2:127–138 27703765 10.1192/bjpo.bp.115.001685 PMC4995584

[39] Liu Z-W, Yu Y, Hu M, et al. PHQ-9 and PHQ-2 for screening depression in Chinese rural elderly. PloS One. 2016;11:e0151042 26978264 10.1371/journal.pone.0151042 PMC4792401

[40] Yu X, Stewart SM, Wong PTK, et al. Screening for depression with the Patient Health Questionnaire-2 (PHQ-2) among the general population in Hong Kong. J Affect Disord. 2011;134:444–447 21665288 10.1016/j.jad.2011.05.007

[41] Xiong N, Fritzsche K, Wei J, et al. Validation of patient health questionnaire (PHQ) for major depression in Chinese outpatients with multiple somatic symptoms: a multicenter cross-sectional study. J Affect Disord. 2015;174:636–643 25576931 10.1016/j.jad.2014.12.042

[42] The Mauco Research Team, Caneo C, Toro P, et al. Validity and performance of the Patient Health Questionnaire (PHQ-2) for screening of depression in a rural Chilean cohort. Community Ment Health J. 2020;56:1284–1291 32193853 10.1007/s10597-020-00605-8

[43] Arrieta J, Aguerrebere M, Raviola G, et al. Validity and utility of the Patient Health Questionnaire (PHQ)-2 and PHQ-9 for screening and diagnosis of depression in rural Chiapas, Mexico: a cross-sectional study. J Clin Psychol. 2017;73:1076–1090 28195649 10.1002/jclp.22390 PMC5573982

[44] American Psychiatric Association. Practice Guideline for the Treatment of Patients with Major Depressive Disorder. Third Edition. Edited by Gelenberg AJ, Freeman MP, Markowitz JC, et al. Arlington, VA: American Psychiatric Association, 2010

[45] Karliner LS, Napoles-Springer AM, Schillinger D, et al. Identification of limited English proficient patients in clinical care. J Gen Intern Med. 2008;23:1555–1560 18618200 10.1007/s11606-008-0693-y PMC2533382

[46] Ryan C. The Limited English Proficient Population in the United States. migrationpolicy.org. Published July 7, 2015. Available at https://www.migrationpolicy.org/article/limited-english-proficient-population-united-states Accessed 34, 2018

[47] California Health Interview Survey. CHIS 2013. Los Angeles, CA: UCLA Center for Health Policy Research

[48] National Latino and Asian American Study. Massachusetts General Hospital. Available at https://www.massgeneral.org/mongan-institute/centers/dru/research/past/nlaas Accessed 820, 2020

[49] August KJ, Nguyen H, Ngo-Metzger Q, et al. Language concordance and patient-physician communication regarding mental health needs. J Am Geriatr Soc. 2011;59:2356–2362 22091992 10.1111/j.1532-5415.2011.03717.x

[50] Chew LD, Bradley KA, Boyko EJ. Brief questions to identify patients with inadequate health literacy. Fam Med. 2004;36:588–594 15343421

[51] Sarkar U, Schillinger D, López A, et al. Validation of self-reported health literacy questions among diverse English and Spanish-speaking populations. J Gen Intern Med. 2011;26:265–271 21057882 10.1007/s11606-010-1552-1 PMC3043178

[52] Elixhauser A, Steiner C, Harris DR, et al. Comorbidity measures for use with administrative data. Med Care. 1998;36:8–27 9431328 10.1097/00005650-199801000-00004

[53] Huntley AL, Johnson R, Purdy S, et al. Measures of multimorbidity and morbidity burden for use in primary care and community settings: a systematic review and guide. Ann Fam Med. 2012;10:134–141 22412005 10.1370/afm.1363 PMC3315139

[54] Sorkin DH, Murphy M, Nguyen H, et al. Barriers to mental health care for an ethnically and racially diverse sample of older adults. J Am Geriatr Soc. 2016;64:2138–2143 27565017 10.1111/jgs.14420 PMC5937991

[55] Mackenzie CS, Pagura J, Sareen J. Correlates of perceived need for and use of mental health services by older adults in the collaborative psychiatric epidemiology surveys. Am J Geriatr Psychiatry. 2010;18:1103–1115 20808105 10.1097/JGP.0b013e3181dd1c06 PMC2992082

[56] Fiscella K, Franks P, Doescher MP, et al. Disparities in health care by race, ethnicity, and language among the insured: findings from a national sample. Med Care. 2002;40:52–59 11748426 10.1097/00005650-200201000-00007

[57] Bibbins-Domingo K. This time must be different: disparities during the COVID-19 pandemic. Ann Intern Med. 2020;173:233–234 32343767 10.7326/M20-2247 PMC7192360

[58] Le TK, Cha L, Han H-R, et al. Anti-Asian Xenophobia and Asian American COVID-19 disparities. Am J Public Health. 2020;110:1371–1373 32783714 10.2105/AJPH.2020.305846 PMC7427240

[59] Kirmayer LJ, Groleau D, Guzder J, et al. Cultural consultation: a model of mental health service for multicultural societies. Can J Psychiatry Rev Can Psychiatr. 2003;48:145–153 10.1177/070674370304800302 12728738

[60] Takeuchi DT, Zane N, Hong S, et al. Immigration-related factors and mental disorders among Asian Americans. Am J Public Health. 2007;97:84–90 17138908 10.2105/AJPH.2006.088401 PMC1716230

[61] Aguilera A, López SR. Community determinants of Latinos' use of mental health services. Psychiatr Serv. 2008;59:408–413 18378840 10.1176/appi.ps.59.4.408 PMC4167056

[62] Nguyen D. Acculturation and perceived mental health need among older Asian immigrants. J Behav Health Serv Res. 2011;38:526–533 21598084 10.1007/s11414-011-9245-z

[63] Alegría M, Roter DL, Valentine A, et al. Patient-clinician ethnic concordance and communication in mental health intake visits. Patient Educ Couns. 2013;93:188–196 23896127 10.1016/j.pec.2013.07.001 PMC3800470

[64] Roter DL, Erby LH, Adams A, et al. Talking about depression: an analogue study of physician gender and communication style on patient disclosures. Patient Educ Couns. 2014;96:339–345 24882087 10.1016/j.pec.2014.05.006 PMC4145035

[65] Adams A, Realpe A, Vail L, et al. How doctors' communication style and race concordance influence African-Caribbean patients when disclosing depression. Patient Educ Couns. 2015;98:1266–1273 26319363 10.1016/j.pec.2015.08.019

[66] Ghods BK, Roter DL, Ford DE, et al. Patient–physician communication in the primary care visits of African Americans and Whites with depression. J Gen Intern Med. 2008;23:600–606 18264834 10.1007/s11606-008-0539-7 PMC2324146

[67] Keller AO, Gangnon R, Witt WP. The impact of patient-provider communication and language spoken on adequacy of depression treatment for U.S. women. Health Commun. 2014;29:646–655 24147987 10.1080/10410236.2013.795885 PMC3991757

[68] Ell K. Depression care for the elderly: reducing barriers to evidence-based practice. Home Health Care Serv Q. 2006;25:115–148 16803741 10.1300/J027v25n01_07 PMC1501087

[69] Colligan EM, Cross-Barnet C, Lloyd JT, et al. Barriers and facilitators to depression screening in older adults: a qualitative study. Aging Ment Health 2020;24:341–348 30588845 10.1080/13607863.2018.1531376

